# Graded Epidural Anesthesia for Non-cardiac Surgery in the Prone Position in a Patient With Low Ejection Fraction

**DOI:** 10.7759/cureus.24685

**Published:** 2022-05-03

**Authors:** Mamta Jain, Nitu Yadav, Anish K Singh

**Affiliations:** 1 Anesthesia, Pandit Bhagwat Dayal Sharma Post Graduate Institute of Medical Sciences, Rohtak, IND

**Keywords:** adverse cardiac events, myocardial ischemia, anesthetic goals, percutaneous nephrolithotomy, coronary artery disease

## Abstract

Ischemic heart disease (IHD), also known as coronary artery disease, occurs due to the blockage of coronary arteries which reduces the blood supply of the myocardium. The main goal of the anesthetic management of IHD patients undergoing non-cardiac surgery is to maintain the balance between myocardial oxygen supply and demand. Here, we report the anesthetic management of an IHD patient with a low ejection fraction who was posted for percutaneous nephrolithotomy in the prone position. We opted for graded epidural anesthesia with a low dose of a local anesthetic drug and opioid. Graded epidural anesthesia is a safe alternative over general anesthesia for patients with IHD and low ejection fraction as it reduces stress response to surgery, provides good postoperative analgesia, and avoids myocardial depressant drugs and coagulation responses.

## Introduction

Patients with ischemic heart disease (IHD) and low ejection fraction (EF) are at risk of intraoperative adverse cardiac events including cardiac arrest. Anesthetic goals in these patients are to maintain forward flow by ensuring normovolemia, decreasing afterload, and avoiding myocardial depressant drugs. Certain surgeries such as percutaneous nephrolithotomy (PCNL) are performed in the prone position, which results in significant changes in the systolic and diastolic functions of the heart, as well as dyssynchrony in cardiac patients [1]. Therefore, anesthetic management tailored to the needs of these patients requires expertise, careful assessment, optimization, and planning the type of anesthesia for cardiac or non-cardiac surgery [2]. Here, we report the case of successful intraoperative anesthetic management of a patient with IHD and low left ventricular EF (30-35%) who was scheduled to undergo left-sided PCNL in the prone position for renal stone disease using graded epidural anesthesia with titrated low doses of bupivacaine and fentanyl. Appropriate consent was taken from the patient for publishing this case.

## Case presentation

A 47-year-old man, weight 80 kg, height 175 cm, and American Society of Anesthesiologists (ASA) physical status III, was posted for left-sided renal stone removal surgery. He was a known case of IHD for 15 years. No invasive coronary intervention was done at that time. He discontinued his medications for heart disease two years back. He was a chronic smoker and had a history of substernal pain after eating and dyspnea on mild physical exertion (New York Heart Association (NYHA) class III). The patient was started on tab. aspirin plus atorvastatin, tab. bisoprolol, tab. ramipril, tab. nicorandil, tab. trimetazidine, tab. pantoprazole, and tab. spironolactone. His substernal pain after eating subsided, and his dyspnea improved from NYHA class III to II on oral medications. On pre-anesthetic assessment, the patient was conscious, oriented, had a heart rate of 70 beats/minute, had a blood pressure of 148/100 mmHg, and his saturation was 96% on room air. On auscultation, an ejection systolic murmur was heard in the apical area. His breath-holding time was 20 seconds. On airway examination, he had Mallampati grade IV, irregular dentition, and a short neck. His basic hematologic and biochemical parameters were largely within normal limits, as shown in Table 1.

**Table 1:** Basic hematologic and biochemical laboratory parameters of the patient. TLC- total leukocyte count; INR- international normalized ratio

Parameters	Patient values
Hemoglobin	14 g/dL
Total leukocyte count	9.3 × 10^3^/µL
Platelets	240,000/µL
International normalized ratio	0.8
Blood urea	24 mg/dL
Serum creatinine	1.1 mg/dL

His chest X-ray showed cardiomegaly and increased bronchovascular markings. An electrocardiogram (ECG) revealed left ventricular hypertrophy with a strain pattern. Echocardiography of the patient showed left ventricular EF of 30-35%, mild mitral regurgitation, and left ventricular diastolic dysfunction II/IV. He had a left-sided renal stone measuring 8 × 4 mm. High-risk consent was obtained explaining all intraoperative risks pertaining to the cardiac status of the patient.

On arrival in the operating room, routine monitors were attached such as ECG, noninvasive blood pressure (NIBP), and oxygen saturation probe, and baseline hemodynamic parameters were recorded. Under aseptic precautions, large-bore intravenous access was secured. Left-sided radial artery catheterization was done under local anesthesia for continuous invasive blood pressure (IBP) monitoring. Cleaning and draping of the patient’s back were done, and an epidural catheter was inserted at the T11-T12 level. Epidural test dose was given using 3 mL of 2% lignocaine with adrenaline to check the correct placement of the catheter. After ruling out the intrathecal and intravascular placement of the catheter, the patient was placed in a prone position. Graded epidural doses of 0.5% bupivacaine (3-5 mL after every 10 minutes) with 5 µg fentanyl were administered to achieve T6 sensory block dermatome level under vigilant monitoring. A total of 9 mL of 0.5% bupivacaine and 50 µg fentanyl were required to achieve this level, following which surgery was started. Oxygen was supplemented through a venturi mask. Noradrenaline infusion at a minimal rate of ≤5 µg/minute was started to maintain blood pressure intraoperatively. Total blood loss was 100 mL. Fluid therapy was administered using a balanced salt solution. Overzealous fluid transfusion was avoided. The rest of the surgery went uneventful. The patient was shifted to the post-anesthesia care unit (PACU) and noradrenaline was titrated according to blood pressure. After two hours, noradrenaline was tapered off and stopped. The patient was shifted from the PACU to the ward with stable hemodynamic parameters.

## Discussion

As the incidence of IHD is increasing, we are encountering more cardiac patients coming for non-cardiac surgeries nowadays [3]. Intraoperative management of such patients is a challenge for anesthesiologists as these patients are at an increased risk for intraoperative myocardial ischemia, infarction, arrhythmias, and congestive heart failure [4]. According to the American Heart Association, normal left ventricular EF is 50-75%. A borderline EF is between 41% and 50%, with less than 35% defined as severely low EF. The main anesthetic goals for patients with low EF include maintaining stable hemodynamic parameters, optimizing oxygen supply to prevent myocardial ischemia, maintaining normothermia, and treating arrhythmias if it develops [5].

In general anesthesia (GA), laryngoscopy and endotracheal intubation lead to sympathetic stimulation causing hypertension and tachycardia. The use of anesthetic gases and intravenous agents can lead to hypotension. During GA, positive pressure ventilation leads to hypoxemia, acidosis, decreased venous return, and hypocarbia or hypercarbia [6]. Regional anesthesia has the advantage of avoiding airway instrumentation. Spinal anesthesia can cause sudden hypotension due to sympathetic blockade. In epidural anesthesia, incremental low doses of local anesthetic drugs avoid hypotension. Moreover, epidural reduces afterload, thus increasing left ventricular forward flow and cardiac output [7]. It provides postoperative analgesia and decreases stress response to surgery, thus decreasing the incidence of myocardial ischemia and arrhythmias. Stress response to surgery affects coagulability by increasing the circulating levels of cytokines and endocrine hormones leading to vascular thrombosis and cardiac morbidity [8]. On comparing systemic opioids and postoperative epidural analgesia in aortic surgery patients, it was found that a reduction in the risk of myocardial infarction with unchanged mortality risk was seen by postoperative epidural analgesia [9].

Major anesthetic concerns in our patient were low left ventricular EF (30-35%), change in position from supine to prone, and maintenance of hemodynamic parameters in the prone position. The prone position causes compression of abdominal vasculature leading to a decrease in the arterial filling and stroke volume, increasing heart rate and peripheral vascular resistance [10]. Further, there is a risk of endotracheal tube dislodgement (in the case of GA), brachial plexus injury, pressure injuries to the eyes and face, and necrosis of dependent areas [11]. Our patient was awake and received a local epidural drug after we positioned him in the prone position, avoiding the above-mentioned complications. Anesthetic goals were to maintain cardiac output, improve oxygenation, and prevent cardiac decompensation. Low and graded doses of a local anesthetic drug with fentanyl were administered while continuously monitoring heart rate, respiratory rate, IBP, and saturation. Minimally invasive blood pressure monitoring should be used in these cases if available. Fluid overload leads to pulmonary edema and cardiac impairment in such patients [12]. Overzealous use of fluid was avoided. Noradrenaline increases the venous return by raising vascular tone in capacitance veins, increasing contractility, and increasing afterload [13]. However, these effects are short-lived, and thereafter it only increases afterload. As epidural injection of local anesthetic drugs also causes hypotension due to sympathetic nervous system blockade, graded doses were given, and noradrenaline infusion was started at a lower dose for a short duration to maintain blood pressure by counteracting the fall in systemic vascular resistance [14] and preload [15] by epidural anesthesia.

Noradrenaline was titrated and tapered off in the postoperative period on recovery of peripheral vascular tone. Because it has been found that postoperative epidural analgesia decreases myocardial injury in IHD patients [16], we administered epidural top-ups of 0.0625% bupivacaine with fentanyl 1 µg/mL in the postoperative period to relieve pain and maintain cardiac stability.

## Conclusions

Graded epidural with slow and guided blockade of dermatomes is a good choice for anesthetizing cardiac patients with low EF, provided the surgery can be done under regional anesthesia. It reduces afterload, decreases stress response to surgery, avoids airway instrumentation and hemodynamic response of laryngoscopy, improves coagulation, reduces blood loss, and provides postoperative analgesia, thus reducing morbidity in IHD patients. This case highlights the benefits of graded epidural and various perioperative considerations in cardiac patients with severe systolic and diastolic dysfunction undergoing non-cardiac surgery in the prone position.
